# Monitoring, Managing, and Communicating Risk of Harmful Algal Blooms (HABs) in Recreational Resources across Canada

**DOI:** 10.1177/11786302211014401

**Published:** 2021-05-05

**Authors:** Hamidreza Rashidi, Helen Baulch, Arshdeep Gill, Lalita Bharadwaj, Lori Bradford

**Affiliations:** 1 School of Environment and Sustainability, University of Saskatchewan, Saskatoon, SK, Canada; 2 School of Public Health, University of Saskatchewan, Saskatoon, SK, Canada

**Keywords:** Harmful algal blooms, environmental scanning, blue-green algae surveillance, risk communications, environmental monitoring, Canada

## Abstract

Globally, harmful algal blooms (HABs) are on the rise, as is evidence of their toxicity. The impacts associated with blooms, however, vary across Nation states, as do the strategies and protocols to assess, monitor, and manage their occurrence. In Canada, water quality guidelines are standardized nationally, but the management strategies for HABs are not. Here, we explore current strategies to understand how to better communicate risks associated with HABs to the public. Our team conducted an environmental scan on provincial and territorial government agency protocols around HABs. Results suggest that there are variations in the monitoring, managing, and communicating of risk to the public: British Columbia, Manitoba, New Brunswick, and Quebec have well-established inter-agency protocols, and most provinces report following federal guidelines for water quality. Notably, 3 northern territories have no HABs monitoring or management protocols in place. More populous provinces use a variety of information venues (websites, social media, on site postings, and radio) to communicate risks associated with HABs, whereas others’ communications are limited. To induce more collaboration on HABs monitoring and management and reduce the associated risks, creating a coherent system with consistent messaging and inter-agency communication is suggested.

## Background

Cyanobacterial blooms are a global concern because of their ecological impacts and potential for toxic effects on human and animal populations.^1–3^ The negative impacts of blooms have led to the adoption of the term “harmful algal blooms” (HABs) reflecting the potential for nuisance, noxious, or toxic impacts of these biomass accumulations.^4,5^ HABs have negative impacts on economies via increased water treatment costs, decreased tourism activities, and closure of water-related industries.^6,7^ HABs affect drinking water and wastewater treatment plants by requiring additional pre-treatment or treatment activity; or in extreme cases, by plant closures as a result of treatment failure.^8–10^

Within freshwaters, cyanobacteria (also termed blue-green algae), are a major concern. Although blooms have been reported under a broad range of environments,^11^ cyanobacteria are most likely to proliferate under warm, calm, and nutrient-rich conditions.^12,13^ Increased populations, and agricultural nutrient usage since the mid-20th century have contributed to eutrophication and worsening HABs.^14,15^ Climatic change effects such as increased water temperatures and changes in the physical structure and phytoplankton communities of lakes have also contributed to increased HABs frequency.^16–19^

A large number of cyanobacterial taxa exist, yet only a subset of these taxa have the potential to produce toxins.^1,13,20^ Whilst the presence of a toxin-producing taxa is concerning, the induction of toxin production is difficult to predict,^20^ so the presence of a species does not equate to bloom toxicity.^4^ Also of note is the wide variety of toxins produced by cyanobacteria, with varied modes of action and health effects on humans which can include fever, nausea and vomiting, rashes and other skin irritations, and gastroenteritis.^21^ The global focus has been on microcystin monitoring due to its prevalence and potential for serious harm.^6,22,23^ Although there is growing concern and indeed calls to monitor and regulate for other toxins, such as anatoxin, guidance and regulations for a wider variety of toxins have only been included in monitoring regulations in a few countries.^24^ Fortunately, cases of illness associated with HABs remain relatively rare; however, the occurrence of HABs and associated illnesses have been increasing in frequency and intensity in the past 30 years. This trend is expected to continue,^25,26^ leading to increased awareness of the need to prepare for HABs and associated threats to public safety.^6,27^ There are calls, globally, to enhance risk communications for a variety of water-related health risks such as algal blooms, chemical toxicants, and infectious agents, especially given enhanced bloom risk anticipated with climate change.^28^

### The Canadian context

Canada is no exception to increased HAB risk, with reports of increasing bloom frequency.^6^ Toxic HABs were first detected in the prairie provinces’ water bodies in the 1940s and 1950s.^29–31^ Since the mid-1990s, monitoring and public awareness of HABs in waterbodies have increased, as has public reporting of impacted water bodies.^29,32^ HAB reports by responsible authorities (eg, local health agencies) are an important part of news and weather bulletins.^33^ There are no definitive annual statistics on the number of HABs in Canada, however media records show that nearly 150 HABs were reported in waterbodies in 2018.^13^ The highest number of waterbodies impacted by HABs in 2018 was observed in Ontario, followed by Alberta, and Manitoba, respectively.^13^ While the other provinces experienced smaller number of HABs in their waterbodies, reports are not consistently investigated or tracked over time. Since 2004, only 8 HABs events were reported in Prince Edward Island, which is the lowest among the provinces.^13,29^ There are no records of HABs in Canada’s 3 territories from 2014 to 2019.^13^

Guidelines for recreational water quality have been standardized nationally. Health Canada established the first edition of the Guidelines for Canadian Recreational Water Quality in 1983,^34^ based on the National Technical Advisory Committee’s (NTAC) recommendations.^35^ These guidelines were revised by the Federal-Provincial and Territorial (FPT) Committee Working Group on Recreational Water Quality respectively in 1992 and 2012.^34^

Recent studies have shown that when guidelines for human health protection related to waterborne risks are considered at the watershed scale, there is better adherence and lower health risks.^36,37^ In Canada, the guidelines produced by the Federal–Provincial Advisory Committee on Environmental and Occupational Health, state that water used for recreation should be free from chemical, physical, and microbiological hazards to guarantee that there is insignificant risk to users’ safety.^38^ According to the guidelines, the values for cyanobacteria and their toxins in recreational waters should not exceed more than 100 000 cells/mL and 20 µg/L for total cyanobacteria and total microcystin, respectively. Higher values have been shown to be harmful to human health.^34,38^ These guidelines were established by a federal committee with representatives from each province and territory, and are meant to follow precautionary principles, however, it is recognized that some waterborne risks only exist temporally and are thereby difficult and not cost-effective to monitor for continuously.^39^

Bloom monitoring varies across Canadian provinces and territories, affecting agencies’ abilities to determine when guidelines are exceeded. When a bloom is experienced, responses to bloom conditions also vary across the country.^13,40^ The increased frequency and inconsistent of bloom responses across Canada is gaining federal notice.^41^ Federal and provincial responses to growing bloom reports, and apparent bloom risk evolved in a punctuated and localized way as water quality awareness grew. Ultimately, there is need for an effective guiding strategy for the management of HABs, and communications about HAB risks to the public for Canadian recreational waters. Indeed, HAB risk communication plans are essential to build public capacity and awareness at the provincial and federal level across Canada.^42^ The implementation of bloom risk management and communications plans provide protection against HABs-related issues for recreational water users.^43^ While none have emerged nationally, some regions like the Great Lakes region have a Bloom Risk Management Program (BRMP) in place. Other regions of the country are lacking in plans; for instance, although Saskatchewan follows the federal guidelines regarding HABs occurrence in waterbodies, there is no formal BRMP at the provincial level.^29^ This is a disadvantage since provincial BRMPs can respond to specific HABs along with providing routine monitoring, sampling, and advisory procedures based on HAB detection at selected sites.^29^ In addition, in the absence of a national bloom risk communication plan, or standardized communication methods to respond to HAB occurrence, Canadian provinces follow different risk communication plans to inform the public.

According to HABs experts, there are international examples of regional, national, and transboundary success in terms of managing, monitoring, and implementing collaborative protocols.^44^ While there are few databases on freshwater HABs, the most accessible source is the Harmful Algal Events Database (HAEDAT). The HAEDAT system is established within the “International Oceanographic Data and Information Exchange” (IODE) of the “Intergovernmental Oceanographic Commission” (IOC) of UNESCO, and in cooperation with the World Register of Marine Species (WoRMS), International Council for the Exploration of the Sea (ICES), North Pacific Marine Science Organization (PICES), International Atomic Energy Agency (IAEA), and International Society for the Study of Harmful Algae (ISSHA).^45^ A collaborative effort between global organizations and member countries established a database, protocols for risk communications, and a scientific program based on consensus approaches to decision making on the over 3000 reported HABs a year managed by these groups.^46,47^ This collaborative’s recognition that HABs occurrence have significant socio-economic consequences on public health, ecosystem health, and recreational activities led to the alignment of monitoring and management procedures in different countries.^48^

North American freshwater monitoring agencies have been slower to create collaborations of a similar nature, but several studies have been conducted in the United States (U.S.) to demonstrate potential effects on socio-ecological systems.^46,49^ Recent studies have demonstrated that uncontrolled algal blooms in freshwaters like Lake Erie could cost billions of dollars over the next 30 years, and that efforts to control these risks could halve these costs.^7^ Given the impact of HAB occurrence in transboundary lakes such as Lake Erie, Lake Champlain-Missisquoi Bay, and Lake Memphremagog, regulations and legislations to reduce impacts require transboundary cooperation, and input from researchers across disciplines within social, ecological, economic, and political sciences, as well as citizens. Since 1909, when the Boundary Waters Treaty (“BWT”) was signed between the U.S. and Canada, ongoing commitments to work together on projects that are of mutual relevance have been respected. In 1970s the first Great Lakes Water Quality Agreement (“GLWQA”) was signed between these 2 countries. According to this agreement, any activities leading to HABs in the Great Lakes is jointly controlled and monitored.^50,51^ International Joint Commission (IJC) between U.S. and Canada provides them with stronger and more focused leadership in terms of addressing HAB-related issues. The commission recommendations include: (a) to strengthen government efforts, (b) to improve existing governance mechanisms, (c) to understand nutrient inputs and outputs, and (d) to develop and initiate implementation of basin-specific action plans.^52^

Given the importance globally of the freshwater resources in Canada, and the growing number of HABs, investment in consistent monitoring and management nationwide is of health, environment, and economic interest, especially given the inter-provincial and inter-territorial nature of freshwaters in Canada.

An understudied aspect of HAB monitoring and management is communication strategies for sharing health risk messages.^34^ Both effective monitoring programs and effective communication strategies are needed to raise public awareness about HABs, promote better management processes in order to recognize and predict issues, understand effective messaging strategies, and reduce negative effects of HABs in freshwater environments.^53^ Thus, the goals of this current study were two-fold:

1. To explore the variations in strategies and protocols used across Canada to assess, monitor, communicate, and manage the occurrence of HABs; and,
2. To suggest ways of enhancing current monitoring and risk communication plans for HABs nationally.

## Methodology

The study used an environmental scanning method to examine the current policies and protocols across Canada related to HABs at both the provincial/territorial and federal scales.^54,55^ The environmental scanning method,^56–58^ a systematic exploration method with formal searching protocols,^59,60^ has widespread application in the fields of environmental management and public policy.^61^ This method is widely applicable in studying persistent health or environmental challenges and trends for their management, as well as new ideas for obtaining information about specific strategies used to solve similar problems in other contexts or jurisdictions.^62^ Environmental scanning is also a principal technique used by researchers and organizations to examine internal and external organizational environments, cultures, and trends that direct how a firm adapts to new needs.^63^ Consequently, this method applies in environmental-related fields such as surface and ground water resource management.^54,63,64^ In the context of environmental health, environmental scanning is an effective method to get a better understanding of how different organizations manage emerging challenges which can threaten public health, such as air pollution and contamination of drinking water.^65^

The environmental scan took place from April 2018 to November 2019 and was conducted by a research team which included 2 graduate students, a postdoctoral fellow, and a faculty member. The work is part of a larger funded program examining HABs formation and risk communications across Canada.

The team scanned agencies in 10 provinces and 3 northern territories for HABs-related acts, legislation, agencies, and practices that were actively involved in HABs management. Web-based searches of current and archived organizational policies and other documents related to HABs control measures occurred, as well as telephone conversations with each agency with responsibilities related to HABs or nutrient monitoring. The National Library of Canada Archives were scanned for historical documents about HABs management. Finally, findings were verified with agencies to ensure accuracy during the study period. Next, findings were compared geographically across the federal system using a map. Additionally, HABs monitoring and control regulations were studied in each province or territory for risk communications strategies.

## Results

The results compile different monitoring and management strategies, and communication methods about HABs across Canada. Results (Table 1) are presented below by governing body (eg, province, territory, and their local authorities). Alongside these results, we list recently reported HABs in each governing area, and actions taken when HABs are present. Table 1 presents an overview of province-by-province and territorial results on governing bodies, structure, and policies. Each region is then expanded in qualitative detail below.

**Table 1.** Overview of provincial HABs risk communication responsibilities.^29,34,42^

Province	Governing body	Current monitoring	Governing policy
Alberta (AB)	Alberta health services (AHS)	Five zones based on population, land mass, and public service provision through blue-green algae/cyanobacteria monitoring program	Federal: Guidelines for Canadian recreational water quality
British Columbia (BC)	Ministry of environment	Works in collaboration with municipalities, and public and private beach owners and operators within 6 different health authorities of the province	Federal: Guidelines for Canadian recreational water quality
			Provincial: Decision protocols for cyanobacterial toxins in British Columbia drinking water and recreational water
Manitoba (MB)	Department of conservation and climate, sustainability development	Manitoba sustainable development; as well as Manitoba health, and seniors and active living	Federal: Guidelines for Canadian recreational water quality
			Provincial: Clean beaches program
New Brunswick (NB)	Department of health (DH)	Department of health (DH) in association with local health advisors and the department of environment and local government (DELG) are responsible for monitoring, sampling, analyzing, and controlling	Federal: Guidelines for Canadian recreational water quality
			Provincial: DH’s guidance for public advisories on cyanobacterial blooms in recreational water
Newfoundland and Labrador (NL)	Department of environment and climate change (DECC)	Under the 1986 Canada-Newfoundland and Labrador water quality monitoring agreement (WQMA), environment and climate change Canada, and the provincial department of environment and climate change conservation monitor ambient surface water quality across the province	Federal: Guidelines for Canadian recreational water quality
Nova Scotia (NS)	The environmental health and food safety division of Nova Scotia environment (EHD)	The Environmental Health and Food Safety Division of Nova Scotia Environment (EHD) in associated with Nova Scotia lifeguard service (NSLS) monitor recreational waters bodies across the province	Federal: Guidelines for Canadian recreational water quality
Ontario (ON)	Public health units and municipalities	Public health units and municipalities are monitoring most of the recreational waters in the province under supervision of the ministry of environment and conservation and parks (MECP)	Federal: Guidelines for Canadian recreational water quality
			Provincial: 12-point response plan
Prince Edward Island (PE)	The department of communities, land and environment, and the department of public health	The monitoring, sampling, and analysis are carried on case by case-basis and the advisory is issued only when there is excessive growth of cyanobacteria in the waterbody	Federal: Guidelines for Canadian recreational water quality
Québec (QC)	The ministry of sustainable development, environment, and action against climate change (MDDELCC)	Environment-Plage Program is the principle provincial guideline developed by the MDDELCC in association with local health departments to monitor 345 beaches across 17 regions	Federal: Guidelines for Canadian recreational water quality
			Provincial: Québec provincial guidelines
Saskatchewan (SK)	Ministry of health and water security agency (WSA)	Since 2012 Saskatchewan started a Healthy Beach Program and 70 recreational sites are listed and monitored under this program	Federal: Guidelines for Canadian recreational water quality
Yukon, Northwest Territories, Nunavut	-	Do not implement routine testing or monitoring at any of recreational sites	Federal: Guidelines for Canadian recreational water quality

### Alberta

The main governing body for health in Alberta is the Alberta Health Services (AHS).^42^ The AHS has 5 different zones, derived by population, land mass, and public services.^66^ The AHS is responsible for monitoring and managing the quality of recreational waters in the province. The AHS follows the federal “Guidelines for Canadian Recreational Water Quality”, as principle legislation, to make decisions about actions to take when there is the presence of cyanobacterial toxins at recreational waterbodies.^29,42^ As of June 2018, the AHS conducts its “blue-green algae/cyanobacteria monitoring program” in 40 public recreational sites, including 30 different lakes with 50 beaches during summer months to monitor for the presence of cyanobacteria and microcystins.^67^ The decision to monitor each site is based on the recreational site’s popularity through visitor counts and water quality parameters.^68^ Additionally, the recreational waterbodies which are not listed on the program are sampled and monitored on a case-by-case basis through public requests or complaints.^67,68^

The AHS is the only responsible body for decisions making and communicating HABs risks to the public. In cases of exceedances of cyanobacterial toxin, the AHS communicates with the public and distributes necessary information through formal communication channels to a variety of responsible bodies including First Nations and Inuit Health Branch (FNIHB), and Alberta Environment and Parks.^67,68^ On federal Indigenous reserve lands within Alberta, the FNIHB is responsible for monitoring and maintaining the quality of recreational waters.^42^ They follow provincial guidelines and report directly to the AHS which shows that in Alberta, both federal and provincial agencies work in collaboration to maintain the overall quality of recreational waters.^42^ Table 2 illustrates the number of HABs that were reported in Alberta’s different zones in 2018 and 2019.^42,67,68^

**Table 2.** HABs formations in Alberta by health zones in 2018 and 2019.^42,67,68^

Zone	Populations (2016)	Land mass (km^2^)	Number of blue-green algae blooms	
			2018	2019
North Zone	484 964	448 500	16	14
Edmonton Zone	1 363 653	11 800	2	1
Central Zone	478 050	95 000	5	2
Calgary Zone	1 622 391	39 300	1	3
South Zone	303 663	65 500	1	1

### British Columbia

In British Columbia, the provincial Ministry of Environment is the main regulatory body responsible for assessing, protecting, and maintaining the quality of water for water uses, including drinking, recreating, agriculture, and for supporting wildlife and aquatic life.^29^ The Ministry of Environment works in collaboration with local municipalities, and public and private beach owners and operators across 6 different regional health authorities.^42^ Similar to the other provinces, BC follows the federal water quality guidelines to manage recreational waters. Waterbodies in different BC regions are also monitored by the Medical Health Officers from health authorities and municipalities.^29^ Generally, municipalities and health authorities monitor beach sites regularly,^69^ however, there are extensive differences in terms of how and when HABs have been monitored through the recreational monitoring program and according to federal and provincial guidelines.^70^

The main provincial guideline in terms of cyanotoxin presence in BC is encompassed in a report titled “Decision Protocols for Cyanobacterial Toxins in British Columbia Drinking Water and Recreational Water”.^71^ Widespread surveillance and monitoring is not performed on a regular basis specifically for cyanobacterial toxins in BC.^29^ Only susceptible waterbodies are sampled, and the HAB-related findings are reported publicly. Desired actions such as closing of recreational sites, issuing of a public advisory, or placing on-site warning signs to protect the health of public are performed by the local Medical Health Officer.^29,42^ The essential findings are communicated to the public, local government, local community groups, and other health authorities via official media.^69,70^

The recreational waters on federal Indigenous reserve land, and unceded Indigenous lands are managed by the First Nations Health Authority, a province-wide sovereign agency that in 2013, took over BC’s First Nation health-related programs, services, and responsibilities from Health Canada’s First Nations Inuit Health Branch—Pacific Region. The authority reports the quality of recreational waters to the Chiefs and Councils of Indigenous communities in BC.^29,42^ At this point, 53 lakes of importance to BC Indigenous communities, with 74 lake sites like beaches or canoe-access points, have been sampled across the province. However, there is a paucity of public reports and announcements on annual HAB occurred in sampling sites in BC overall.^42,69,70^

### Manitoba

In Manitoba, the “Clean Beaches Program” is the main monitoring and protection program with regards to HABs that operates under the provincial Department of Conservation and Climate, Sustainability Development Division, Water Section.^72^ This provincial program has been developed in close collaboration of the Water Quality Management Section of the provincial Department of Sustainable Development; with Manitoba Health, and Seniors and Active Living also contributing.^73^ In this program, personnel regularly monitor almost 60 beaches across the province for the presence of HABs.^29,42^ Monitoring is performed weekly or monthly based on the popularity of recreational spots and samples are sent to Manitoba’s provincially-authorized analytical labs for testing.^73^ Other waterbodies that are not part of this program are inspected when personnel receive a call from the public, regional authorities, and other stakeholders about a possible HAB.^42,74^ The official website of Manitoba’s Department of Sustainable Development, along with local beach websites, Twitter accounts of the Manitoba Government, and physical signage are used for public risk communication.^74^ In the presence of HABs, the Department of Sustainable Development uses 2 types of physical signs to warn the public to avoid any types of contact with water.^29,73^ Moreover, factsheets, posters, and other informative materials are posted on the Department’s website by the Health, Seniors and Active Living, and Sustainable Development’s Water Quality unit for raising public awareness of HABs occurrences.^74^

**Figure 1.**
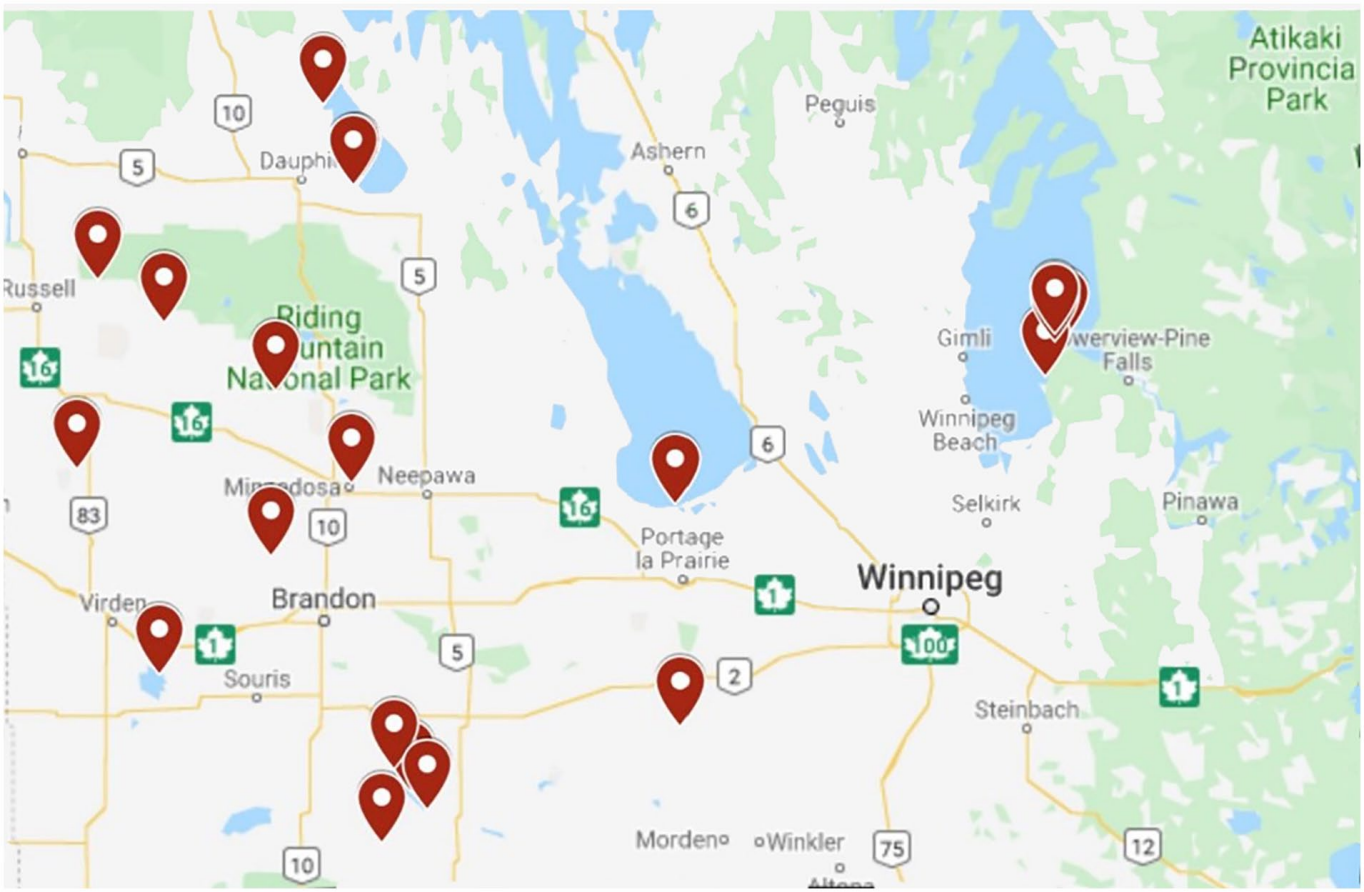
Screenshot of publicly-available map of HABs occurrence across Manitoba in 2019.^73,75^

The Manitoba Department of Sustainable Development marks and updates the affected recreational sites on a provincial map displayed on the Government of Manitoba website. As of September 2018, the map specified 12 waterbodies out of 57 sampled across the province that had experienced blue-green algal blooms in the swimming season to a level surpassing the national guidelines. As shown in Figure 1, 21 beaches across the province were reportedly affected by HABs in 2019.^73^

In Manitoba, although the federal agency FNIHB is primarily responsible for monitoring and managing the quality of recreational waters present on-reserve, monitoring is only completed when a request comes from the Chief or Council members of each reserve.^42,75^

### New Brunswick

New Brunswick, similar to the previous provinces, follows the federally-published guidelines for recreational water quality. The province has no regionally-specific protocols and policies for regular monitoring of the recreational sites. Only a few recreational sites are monitored for water quality and sampled across the province by the provincial Department of Health (DH), the responsible organization in association with local health advisors.^42,76^

In response to public requests, the DH, and the Department of Environment and Local Government (DELG) monitor, sample, analyze, and control for cyanobacteria.^29^ The departments have co-created a protocol, called the Algal Bloom Response Protocol, which is the main guide for reporting algal bloom occurrence as part of official provincial records.^77^ Additionally, the Department of Health has developed a protocol called DH’s Guidance for Public Advisories on Cyanobacterial Blooms in Recreational Water.^76^ This protocol provides instructions on the communication of the advisories with the public about cyanobacterial blooms. The presence of cyanobacteria in recreational waters is communicated to the public online through the official website of Government of New Brunswick, and through public news channels, and signs posted at affected sites.^42^ In both the 2018 and 2019 annual reports, the exceedances of blue-green algae (against federal HAB guidelines) were reported in 17 locations across New Brunswick (Table 3).

**Table 3.** Blue green algae exceedance (against federal HAB guidelines) across New Brunswick in 2019.^77^

Location	Date
Saint John River (Fredericton to Woodstock)	2019-present
Urqhart’s Cove in Belleisle Bay	2019-present
Mactaquac Main Beach	2018-present
Lake Nictau (Mount Carleton region)	2018-present
Nashwaak Lake	2017-present
Wheaton Lake	2015-present
Washademoak Lake	2015-present
Grand Lake	2015-present
Harvey Lake	2015-present
Camp Lake	2015-present
Bathurst Lake	2015-present
Chamcook Lake	2010-present
Lac Unique	2010-present
Irishtown Nature Park	2010-present
McLaughlin Reservoir	2010-present
Lac Baker	2010-present
Lake Utopia	2008-present

### Newfoundland and Labrador

The provincial Department of Environment and Climate Change (DECC) is responsible for monitoring cyanobacteria in recreational waters and maintaining the quality of waters in Newfoundland and Labrador. The department samples water on demand, and on-site examination is done on a case-by-case basis when they receive reports of algal blooms.^29,42^ The department follows the federal guidelines for monitoring and taking actions. Complete information on the number of reported algal blooms in 2018 and 2019 are unavailable and only 2 main HAB occurrences were reported on governmental websites.^78^ If there is indication of HABs presence at the recreational site, the information is communicated to the public through on-site signage, and the official provincial government website, local news sites, and local social media platforms.^29,78^

### Nova Scotia

The provincial Environmental Health and Food Safety Division of Nova Scotia Environment (EHD) of Nova Scotia is the responsible organization for monitoring and controlling the levels of bacteria and blue-green algae in recreational waters in the province.^79^ Working tightly with the Nova Scotia Lifeguard Service (NSLS), the 2 agencies provides on-site surveillance of recreational waters across the province. The personnel of NSLS conduct sampling of 23 supervised beaches and interprets the results in association with EHD.^29,80^ Similar to the other provinces, Nova Scotia follows the federal guidelines for monitoring and management.^29^ When toxin levels exceed the recommended federal levels, EHD informs the Regional Medical Officer of Health who takes action.^42,81^ The action includes 3 steps: (a) closure of contaminated beach or recreational site, (b) informing lifeguards and other personnel, and (c) posting information on official website of NSLS about beach’s closing/re-opening schedule. In addition to the NSLS, the Halifax Regional Municipality monitors and maintains the quality of all recreational waters located in the city’s recreational areas in collaboration with Halifax Water, Nova Scotia Environment, and the Regional Medical Officer.^29,42,81^ Although the municipality takes the action, it only monitors beaches where lifeguard supervision is provided. The findings are communicated to the public through on-site signage and on duty-lifeguards at affected locations, social media, official website crawlers, and a recreational beaches’ hotline.^29,34,81^ However, there are no public reports released on annual HAB occurrence at Nova Scotia’s beaches and lakes. In 2018, blue-green algae were reported in at least 3 lakes.^79,81^

### Ontario

Public Health Units and Municipalities in Ontario are the main local authorities which are actively monitoring most of the recreational waters in the province. Working under supervision from the provincial Ministry of Environment and Conservation and Parks (MECP), local units complete monitoring procedures.^82^ Similar to the other provinces, Ontario follows the federal guidelines, but also have their own provincial protocols, called the 12-point Response Plan.^82^ This plan aligns the needs of 7 different water related acts: (a) Great Lakes Protection Act, (b) Environmental Protection Act, (c) Ontario Water Resources Act, (d) Safe Drinking Water Act, (e) Clean Water Act, (f) Nutrient Management Act, and (g) Lake Simcoe Protection Act.^29,42,82^ All responsible agencies receive algal bloom occurrence reports from their weekly regular site visit sampling (during peak algae season) and case-by-case reports from the public.^29^ When HABs are present, the provincial Ministries of Environment, Climate Change, Natural Resources, Conservation Authorities, and local municipalities will take on operational duties.^82,83^ An advisory will be issued based on visual inspection, and further actions such as recreational site or beach closure will be taken after laboratory confirmation of HABs.^29,42^ The exceedance levels of cyanobacteria are communicated to the public through different official channels and social media sites for health unit and provincial government agencies, beach websites, local news sites, and using on-site signage at affected sites. Some health units also increase public awareness of HABs through public education campaigns before the start of swimming season.^82–84^

Like the other provinces, the federal First Nations and Inuit Health Branch is responsible for monitoring of recreational waters on Indigenous lands in Ontario. With the support of Indigenous community-based Environmental Health Officers (EHOs), on-reserve water bodies are visually inspected and sampled.^85,86^ However, monitoring of recreational waters is performed irregularly due to issues of funding, capacity, and access on some reserves and the HABs management varies in each community.^29,42^ In 2018, around 15 Indigenous reserve waterbodies were affected by blue-green algae across the province.^82^

### Prince Edward Island

The Department of Communities, Land, and Environment, and the Department of Public Health are the 2 provincial organizations responsible for monitoring and controlling HABs in recreational waters in Prince Edward Island (PEI).^29^ The PEI government indicates that it follows the federal guidelines for monitoring and management of cyanobacteria in its water bodies, however, there is no regular program for monitoring HABs at recreational sites.^29,42^ The monitoring, sampling, and analyses are carried out on a case-by-case basis and an advisory is issued only when there is a report of excessive growth of cyanobacteria in a waterbody. Any findings are communicated to the public through on-site signage and alerts are posted on the official website of the Government of PEI.^29,42,87^ A lack of annually published reports on HABs in PEI means there are a few statistics to report over time, but since 2004, 11 HABs have been reported in the provincial freshwater bodies through the media. In 2018, 2 HABs occurrences were reported in the province.^88^

### Quebec

The provincial Ministry of Sustainable Development, Environment, and Action Against Climate Change (MDDELCC) is the main official authority tasked with monitoring and controlling HABs in Quebec, where there are more than 400 public water-based recreational sites.^29,34^ The Environment-Plage Program is the principle provincial action strategy developed by the MDDELCC to monitor 345 beaches across 17 regions in the province during swimming season.^89^ By observing and reporting HABs to the MDDELCC and local health departments, the Environment-Plage Program personnel collaborate within the province for sampling and analyses. When a HAB is discovered at a public recreational site, the site is either totally or partially closed. The findings are communicated to the public through on-site signage at affected waterbodies; the official websites of MDDELCC, municipality, and provincial Department of Public Health; and through regional tourist associations and different social media sites.^29,34,89^

The recreational waters on Indigenous reserves in Quebec are monitored by Health Canada’s FNIHB during swimming season. Local action plans are prepared by the FNIHB in collaboration with health directors of each Indigenous community in Quebec. The results of water monitoring samples are communicated to the directors of operations and Chiefs of communities. The results are then communicated to the entire community through on-site signage at the waterbody and relevant social media such as radio.^29,34,42^ Reports are only provided in French language, and there is no data available on the number of blooms reported or observed in 2018 and 2019 in Quebec.

### Saskatchewan

The Saskatchewan Ministry of Health (MOH) and Water Security Agency (WSA) are the provincial organizations that monitor HABs in recreational sites in Saskatchewan.^42,90^ Since 2012, the MOH implemented a Healthy Beach Program under which 70 recreational spots are monitored.^42^ The popular recreational spots are monitored occasionally (some weekly, others monthly or seasonally) from the end of June to the end of August, and other included recreational spots in the Healthy Beach Program are monitored at least once in 5 years.^29^ The public, however, does not have immediate access to HAB monitoring results, and the results are only published a) when public health issues arise, or b) after the summer season has past and technicians have time to report. In the event of a HAB, the findings are communicated to the public through on-site signage at the affected spots, news, and social media.^29,34^ On-site signs samples are emailed to local site supervisors (eg, campground officials and business owners), and the local contact is expected to erect the signage. As of 2018, the official websites of provincial government agencies (MOH and WSA) were used to issue an advisory.^90^ Although, monitoring for HABs is performed regularly, the annual reports have only been shared with the public since September 2020.^91^ No monitoring on Indigenous reserves occurs in Saskatchewan.

### Yukon, Northwest Territories, Nunavut

Because of low number of HABs in Canada’s Northern Territories, the governmental agencies responsible have not yet established a regular monitoring programs for the Territories’ waterways. In the past decade, no exceedances of HABs in water bodies beyond the federal guidelines have been reported by any of the 3 territories.^29,34,42^

### Summary of results

**Figure 2.**
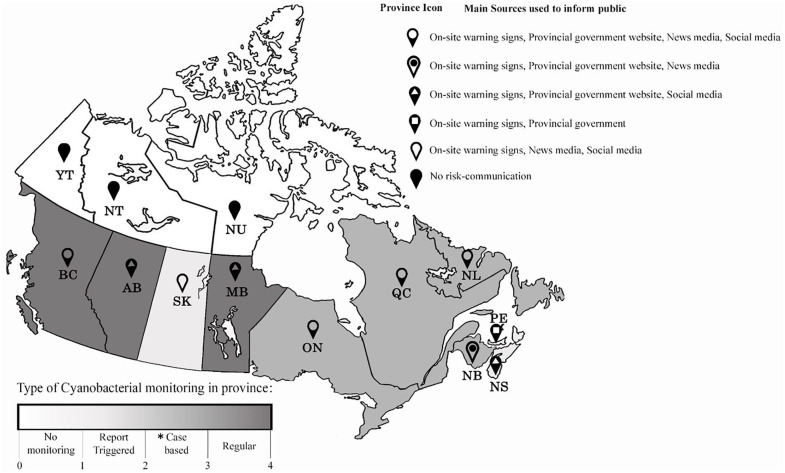
Risk communication on HABs across Canada. *Case-based communications are the reports informing the public at the time of presence of HABs.

While the federal guidelines are being used uniformly across provinces as foundational elements in HABs management strategies, each province has a unique combination of monitoring and management agencies involved and different risk communication strategies for relaying threats about HABs to the public. Figure 2 and Table 4 summarize the results on a map of Canada:

**Table 4.** Departments responsible for HABs monitoring and risk communication.

Provinces and territories	Departments responsible for monitoring and risk communication
Newfoundland and Labrador (NL)	Department of Environment and Climate Change (DECC)
Prince Edward Island (PE)	Department of Communities, Land, and Environment
	Department of Public Health
Nova Scotia (NS)	Nova Scotia Environment
	Nova Scotia Lifeguard Service (NSLS)
	Halifax Regional Municipality
	Regional Medical Officer
New Brunswick (NB)	Department of Environmental and Local Government (DELG)
	Department of Health
Quebec (QC)	Ministry of Sustainable Development, Environment, and Action Against Climate Change (MDDELCC)
Ontario (ON)	Ministry of Environments and Climate Change, and Conversation and Parks
Manitoba (MB)	Manitoba Department of Sustainable Development
	First Nations and Inuit Health Branch on reserve
Saskatchewan (SK)	Ministry of Health (MOH)
	Water Security Agency (WSA)
Alberta (AB)	Alberta Health Services (AHS)
British Columbia (BC)	Ministry of Environment
	Medical Health Officers from Health Authorities
	First Nation Health Authority on Reserve
Yukon (YT)	-
Northwest Territories (NT)	-
Nunavut (NU)	-

The findings reveal some interesting results on the national scale. First, provinces with higher populations (ON, BC, QC, AB) tend to have more agencies involved in HAB management, and some level of established proactive monitoring program. Second, none of the provincial organization with HABs responsibilities described relationships with other government agencies involved in agricultural management, tourism, or veterinary services in their lines of communication. Third, in most provinces, communications on risks of HABs occurred once a confirmation of HAB was received—there was no coordination of HAB awareness campaigns across provincial borders, or through multi-level strategies such as warning the public about HABs hot-spots, past occurrences, or ways of getting in touch with experts. The communications were described as being unidirectional.

## Discussion

Similar to the other regions of the world, the occurrence of cyanobacterial blooms has increased in Canadian water bodies over the past decade.^6^ With increased threats to human and ecosystem health, consistent approaches to monitoring programs nationally for HABs are important.^32,34^ There should also be a greater focus on monitoring in known bloom-affected recreational water bodies. Although variations are found in the monitoring, management, and communications around HABs risks across Canada, all the provinces described following the Federal Guidelines for Canadian Recreational Water Quality, reinforcing the value of federal leadership.^29,34,42^

In Canada, new policies related to HAB occurrences can be regulated by either federal or provincial authorities. Although the federal guidelines provide a comprehensive policy in terms of water quality limits, and recommended HABs related procedures for all the provinces, the provincial regulatory bodies recommend a more active program with public education campaigns to avoid, reduce, address HAB occurrence, and communicate effectively with people about HABs. Some provinces like Ontario, British Columbia, Quebec, New Brunswick, and Manitoba, with their own provincial protocols and regulations, had a more contextualized response to address case-by-case HAB occurrences and issues. Such localized regulations include controlling and monitoring non-point sources of phosphorus and nitrogen.^92^ Non-point sources are the runoff and drainage flows that enter the water bodies such as through ground water, rivers, and lakes (Lake Erie Action Plan in Ontario).^50,51^

Other provincial actions include the regulatory and operational tasks and duties, which authorize municipalities and cities to deliver public awareness campaigns, contribute to capacity building, and promote behavior change to protect recreational waters, water bodies, and local environments.^50^ Generally, municipalities implement the adjusted federal and provincial regulations to local sites where it is essential for public safety (eg, beaches within city limits). The monitoring role of municipalities in Ontario, British Columbia, Quebec, and Nova Scotia had an impact on reducing HAB risks in recreational sites and other water bodies.

In terms of monitoring procedures, the provinces exist currently in 2 main groups: the active managers and the reactive mitigators. While some provinces perform regular monitoring alongside active risk mitigation measures; communicate with the public about observed blooms; and follow their provincially-specific protocols to manage HABs; others perform ad hoc monitoring with little-to-no collaboration among agencies and stakeholders. Provinces such as Ontario, British Columbia, Manitoba, and Quebec have specific protocols and programs for monitoring and managing HABs in waterbodies, which makes sense given their higher observed rates of HABs in the literature, greater recreational opportunities in lakes, and population demographics.^29,71,72,82,89^ Provinces with interagency networks between water, health, and other operational organizations listed a higher level of monitoring and more potential actions in their plans in response to HABs.^50^ In other words, the broad network between different bodies in these provinces supported multiple and varied actions to limit HABs: they could alter the discharge of contaminated municipal sewage by applying developed treatment techniques in wastewater treatment plants (WWTP); recommend strategies to manage stormwaters; and build effective capacity for public reporting of HABs in urban areas through social media-based channels.^50^

Provinces like Alberta, Newfoundland and Labrador, Nova Scotia, New Brunswick, and Saskatchewan implement the federal guidelines as the primary action, with few other actions taken, and only on public reports of HABs.^29,34,42^ Interestingly, and likely due to the low occurrence of HABs and lower populations, the 3 northern territories (Nunavut, Yukon, and Northwest Territories) and Prince Edward Island do not report having any provincial or territorial guidelines for monitoring and managing HABs.^34,42,88^ This is concerning given published reports on how changing agricultural, environmental, tourism, and climatic parameters have been contributing to increased blooms risks in regions previously not affected by cyanobacteria.^93,94^

When comparing the different levels of monitoring of HABs across provinces, only 3 provinces (Alberta, Manitoba, and Quebec) conduct regular proactive monitoring.^67,73,85,89^ Monitoring is triggered by reporting in Ontario,^82^ whereas other provinces perform case-by-case sampling to determine if a bloom is a health threat.^85^ No monitoring is performed in the 3 territories.^85^ The First Nations and Inuit Health Branch is responsible for monitoring on Indigenous reserve lands in collaboration with provincial governments, however, it is reported that different reserves have varied levels of monitoring capacity and communications around HABs.^34,42^

Similar to the variations seen in monitoring and protocols across Canada, there are variations in communicating risks to the public. Four provinces (British Columbia, Newfoundland and Labrador, Ontario, and Quebec) use a wide range of sources such as social media, news media, provincial government websites, beach websites, radio, on-site physical signage, issuing of public advisories, and health informatics to communicate information about HABs and risk associated with them to the public.^29,42,85^ Communication mechanisms are, however, limited in other provinces.^42,88,90^ It is interesting that communications are described as 1-time events, rather than systems of communications which could involve educational campaigns, inter-agency communications, factsheet production, websites, podcasts, and other tools that have been found to be effective for communications about HABs in other nations.^95,96^ Agencies did not list any regional BRMPs that were in place.

Lack of implementation practices, continuing surveillance, and active bloom mitigation and communication with the public about HABs can lead to increase in threats to the health of humans, livestock, and pets.^12,17^ This potential threat makes it important to systematically increase monitoring of waterbodies for the presence of algal blooms and to enhance communication with the public.^6,83^ The creation of a unified program among all the provinces to monitor and communicate about HABs is a possibility given the uptake of the federal guidelines. Collaboration is needed between federal government, provincial governments, FNIHB, and Indigenous communities to establish such a program and build capacities to enact that program across watersheds in Canada and in ways that reflect the nuances of various regions. The Federal Guidelines for Canadian Recreational Water Quality provide the essential principles in terms of cyanobacterial blooms^29^; however, a national program would suggest best practices with flexibility for modifications based on each province’s geographical, demographic, and climatic conditions. An Indigenous strategy co-created with Indigenous communities and federal, provincial, territorial, and other relevant agencies is also needed. In consideration of the environmental, climate, and demographical changes in the northern territories, it is essential to develop specific and localized HAB related policies, prepare needed infrastructure, and build human capacity for interactive monitoring and risk communication among communities whose lives are dependent on northern water bodies. Given the success of international collaborative programs such as HAEDAT^46,47^ and the ICES-IOC Working Group on Harmful Algal Bloom Dynamics,^49^ a national-level collaborative approach in compiling datasets on HABs and sharing best practices for risk communications would be a start.

To protect public health, it is recommended that scheduled and mandatory monitoring of waterbodies across provinces and territories occurs within peak periods of occurrence and recreational usage, utilizing different conventional and innovative techniques such as remote sensing.^6,97^ This can be achieved by further collaboration of provincial governments with environmental monitoring and management agencies locally, provincially, nationally, and internationally; local health authorities; municipalities; and beach owners/management. Sampling, monitoring, and testing can be performed by local health authorities, municipalities, and beach owners, where advisories could also be issued by provincial governments and tourism agencies to a wider public who may use recreational sites but not live in the local region. It is important that the monitoring process be reinforced by an active policy, be designed for a regular time period given its time-span dependent nature, and be implemented using a plan and metrics to ensure long term improvement (eg, Lake Winnipeg Basin (LWB) programs).^54^

The presence of algal blooms and risks associated with them can be communicated to the public by provincial governments through all available sources such as on-site signage, social media, news media, radio, and official websites of government agencies involved in health, environment, natural resources, and recreation. Moreover, online reporting and interactive site mapping (eg, as in Manitoba) can effectively raise public awareness of real-time HABs and related health risks.

There is a great need for the initiation of baseline surveillance of waterbodies located on reserves for the presence of cyanobacteria due to the continuance of traditional lifestyles and economies dependent on hunting and fishing. This can be achieved through meaningful collaboration with communities themselves, the federal government, provincial governments, FNIHB, and tribal councils. It is also notable that traditional practices offered by Indigenous communities around the world including Canada could be integrated into management actions and policies to address HAB occurrence and related issues.^98,99^ In addition to monitoring, there is a need to communicate risks associated with algal blooms in culturally acceptable ways.^100,101^ Engaging all stakeholders, right-holders, and the public in developing HAB-related action plans and policies, and providing publicly accessible HAB data can build trust and facilitate better communication to reduce HABs risks, and related issues in Indigenous communities through to cities (eg, LWB program in Manitoba, Saskatchewan, and Alberta; and Nature Alberta Program in Alberta).^54^ The application of new accessible technologies in HABs-related fields such as smartphone-based nutrient monitoring apps, Swim-Drink-Fish Apps,^102–104^ and public awareness programs in Indigenous communities can be helpful for promoting knowledge-sharing about HABs-related issues.

Given the emergence of collaborative monitoring systems globally that are very successful for both monitoring and reducing negative economic effects of HABs in the oceanic, and freshwater context,^105–108^ a review of Canadian policy at the federal and provincial policies for monitoring for HABs is worthwhile. Results reported here demonstrate that without a nationally coordinated system, provincial disparities in monitoring and inequality of monitoring activity and capacity on reserves leaves populations at risk of HABs-related health effects. In addition, experts have put forward effective communications systems such as HAEDAT^45^ that serve to alert agencies internally, and across jurisdictions, of risk levels allowing for the timely provision of advice to the public. It is clear that there are gaps in services provided across Canada for HABs monitoring and communications which could lead to negative health effects, environmental degradation, and economic costs.

## Conclusions

This research showed that the increased occurrence of harmful algal blooms is a global issue in need of attention from countries with large freshwater resources, including Canada. Currently, there are variations in the policies for monitoring HABs and communicating risks across the different jurisdictions in Canada. Creating a coherent system with consistent messaging and inter-agency communication can reduce risks, enhance public knowledge of HABs occurrence, and induce more collaboration on HABs monitoring and management.

The design and implementation of an integrated communication system for HABs provincially with close collaboration of all stakeholders facilitates risk reduction as demonstrated in the case of Ontario. A first step might be to modify federal guidelines and protocols to meet each province needs and mandate the regular and consistent monitoring of all popular recreational sites regularly using an accessible and interactive HABs database across Canada. Such a combined effort would be of enhanced value to provinces that have transboundary waterways such as Manitoba and Ontario, Alberta and British Columbia; and to researchers to identify trends.

A program such as this should be developed with meaningful engagement of Indigenous community members, FNIHB, and provincial and federal stakeholders. The goal would be to create, test, and implement a coherent tool to effectively monitor and communicate HABs in all communities, including Indigenous ones. Employing new technologies that involve citizen science such as the Nutrient App^104^ along with enhanced public awareness programs can build effective capacity. There is also the need to provide culturally acceptable communication strategies among Indigenous Canadians to ensure communications will be effective to reduce health risks. Working collaboratively to design Indigenous communications systems for HABs is especially important for northern communities where the population trends and recreational water usage are highly varied, and where no agencies have implemented monitoring, management, or risk communications programs for HABs. Yet, the realities of climate change indicate that it would be prudent to start on that path.

### Limitation

This work is not without limitations. Environmental scanning can produce a large volume of information which is difficult to digest. In this case, there were few available examples of complex inter-agency relationships on HABs management and risk communications leaving many questions unanswered. In addition, new strategies for HABs management and risk communications may have emerged since the scan occurred. Each jurisdiction had different strategies, programs, and indicators, resulting in different levels of success. Thus, it is difficult to suggest the best ones to apply across a system. Nevertheless, this study has taken the pulse of Canadian HABs management and risk communications across the devolved units and sought to gain an awareness of current strategies and gaps. The most common approaches for raising awareness of risks were local on-site signage, which was used to various success across the country, and web-based communications, which was relayed as the preferred method by agencies for reporting and the perceived best method for the public to access that information. Future work will test that perception.

We hope this work inspires national level conversations on ways to enhance monitoring, management, and risk communications on HABs in Canada. With growing concern due to increased occurrences, establishing better management and communications practices nationwide is a pressing issue. We also hope this work provides momentum for future studies on the effect of different HAB policies, monitoring, and risk communication strategies on social, economic, and health outcomes in different provinces and territories in Canada. Future studies on new risk communication technologies using innovative and interactive apps will be also worthwhile to address HABs in Canada.
